# Intrinsic Exercise Capacity and Mitochondrial DNA Lead to Opposing Vascular-Associated Risks

**DOI:** 10.1093/function/zqaa029

**Published:** 2020-11-03

**Authors:** Shaunak Roy, Jonnelle M Edwards, Jeremy C Tomcho, Zachary Schreckenberger, Nicole R Bearss, Youjie Zhang, Eric E Morgan, Xi Cheng, Adam C Spegele, Matam Vijay-Kumar, Cameron G McCarthy, Lauren G Koch, Bina Joe, Camilla Ferreira Wenceslau

**Affiliations:** 1 Department of Pharmacology and Physiology, University of Toledo College of Medicine and Life Sciences; 2 Department of Radiology Nationwide Children's Hospital, OH, USA

**Keywords:** vascular physiology, intrinsic exercise capacity, mitochondria

## Abstract

Exercise capacity is a strong predictor of all-cause morbidity and mortality in humans. However, the associated hemodynamic traits that link this valuable indicator to its subsequent disease risks are numerable. Additionally, exercise capacity has a substantial heritable component and genome-wide screening indicates a vast amount of nuclear and mitochondrial DNA (mtDNA) markers are significantly associated with traits of physical performance. A long-term selection experiment in rats confirms a divide for cardiovascular risks between low- and high-capacity runners (LCR and HCR, respectively), equipping us with a preclinical animal model to uncover new mechanisms. Here, we evaluated the LCR and HCR rat model system for differences in vascular function at the arterial resistance level. Consistent with the known divide between health and disease, we observed that LCR rats present with resistance artery and perivascular adipose tissue dysfunction compared to HCR rats that mimic qualities important for health, including improved vascular relaxation. Uniquely, we show by generating conplastic strains, which LCR males with mtDNA of female HCR (LCR-mt^HCR^/Tol) present with improved vascular function. Conversely, HCR-mt^LCR^/Tol rats displayed indices for cardiac dysfunction. The outcome of this study suggests that the interplay between the nuclear genome and the maternally inherited mitochondrial genome with high intrinsic exercise capacity is a significant factor for improved vascular physiology, and animal models developed on an interaction between nuclear and mtDNA are valuable new tools for probing vascular risk factors in the offspring.

## Graphical Abstract

**Figure:**
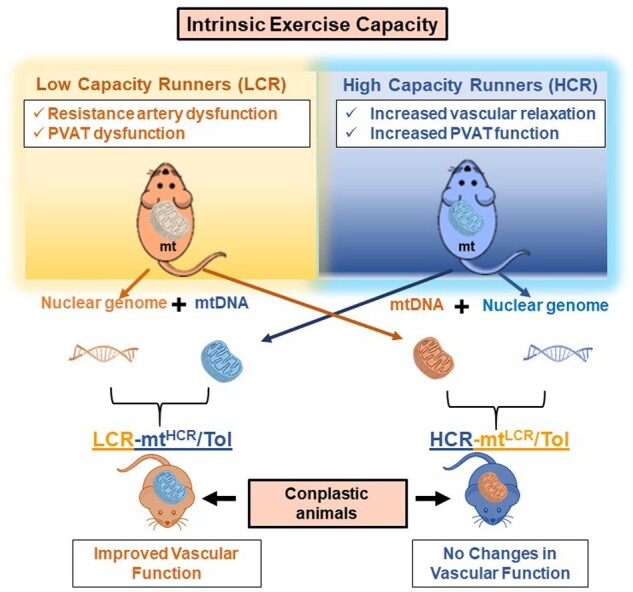

## Introduction

It is well-established that exercise brings about a host of benefits to the health of an individual^1–3^. Near the close of the 20th century, the HERITAGE family study established that our untrained capacity to exercise, or our intrinsic exercise capacity, is genetically heritable^3^. Identifying the factors associated with the heritability of exercise capacity in humans is challenging. Therefore, a large-scale artificial selection for low and high aerobic treadmill-running capacity was performed with genetically heterogeneous N:NIH stock rats. As a result, two divergent rat strains, one with low aerobic running capacity (LCR) and the other with high aerobic running capacity (HCR) were generated.^4^ These strains differ in running capacity by 8.3-fold with an estimated over 40% heritability.^4^ It was also observed that LCR presented with elevated disease risk factors such as higher visceral adiposity, serum-free fatty acid levels, and hyperlipidemia, features consistent with the metabolic syndrome.^5^ On the contrary, HCRs present with qualities important for health, including higher VO_2_max, myocardial function, energy expenditure, and a lean body mass, as well as greater mitochondrial biogenesis.^4^^,^^5^ Since 2005, numerous studies use LCR as a model of disease risk and HCR as a model of a healthy state.^5^^,^^6^ By using animals with contrasting intrinsic exercise capacities, it is possible to determine the exact triggers that lead to cardiovascular abnormalities and the genetically heritable benefits of exercise in untrained animals.

Low intrinsic exercise capacity and physical inactivity lead to cardiovascular diseases.^6^^,^^7^ One of the major pathophysiological characteristics of cardiovascular diseases, such as obesity and metabolic syndrome, is the presence of small artery dysfunction and remodeling.^8–11^ Given that intrinsic exercise capacity, in addition to cardiovascular disease risk, is genetically heritable, we questioned if deleterious vascular plasticity would be present in LCR, while beneficial vascular plasticity would be present in HCR. Our first aim was to determine whether intrinsic (untrained) exercise capacity induces divergent vascular plasticity. Here we observed that the arteries from HCR have increased vascular relaxation and anticontractile effect of perivascular vascular adipose tissue (PVAT). On the contrary, LCR presents vascular atrophy and a procontractile PVAT. By contrast, studies with closely apposed PVAT from HCR and arteries from LCR improved vascular function in arteries from LCR. These data reveal a novel, dichotomous mechanism for vascular homeostasis in inherited exercise capacity.

Humans are the result of billions of years of coevolution between mitochondrial and nuclear genomes. Studies in the 21st century began demonstrating the interplay between these two genomes and how mitochondrial variations can have significant impact on gene expressions and phenotype.^12^ Additionally, mitochondrial DNA (mtDNA) has further emerged as an attractive target to researchers for its potential role in disease susceptibility by also altering mitochondrial function.^13^ Exploring this interplay in humans is difficult, however, breeding conplastic animals enables us to “swap” mtDNA and serves as a powerful tool to enhance our understanding. As we have previously explored this interplay in the hypertensive animal models of Dahl salt-sensitive rat and the spontaneously hypertensive rat,^14^ we decided to initiate a similar breeding scheme with LCR and HCR. Through a series of backcross breedings between LCR and HCR, we generated LCR inbred strains with HCR mitochondria and vice versa. Inheritance of metabolic disease risks in these inbred LCR and HCR models have been previously reported by our group.^15^ Given that mitochondria have a significant impact on vascular physiology,^16^^,^^17^ our second aim was to investigate whether variations in mtDNA would affect vascular plasticity in untrained aerobic running capacity. Here, we provide the first evidence that rats which inherit mtDNA of high intrinsic exercise capacity have improved vascular physiology, while those with mtDNA of low intrinsic exercise capacity present with vascular dysfunction.

## Materials and Methods

### Animals

LCR and HCR rats were generated from genetically heterogenous N:NIH stock.^1^^,^^4^ The development of high‐ and low‐capacity runner rats (HCR and LCR) displaying high and low intrinsic exercise capacity has been described previously.^1^^,^^4^ Generation 26 of these strains has been cataloged in the rat genome database (RGD; https://rgd.mcw.edu/rgdweb/report/strain/main.html?id=10402167; https://rgd.mcw.edu/rgdweb/report/strain/main.html?id=10402163). In this study, we used male LCR and HCR rats (20–26-weeks-old) from generation 42 of selection; male LCR-mt^HCR^/Tol and male HCR-mt^LCR^/Tol Toledo strain (Tol) rats (30–36-weeks-old) (University of Toledo, Toledo, OH, USA). Given that the selectively bred rats present with a background genetic variability which could impact the interpretation of the data, we also performed a key experiment (vascular function), which is pertinent to this study, in arteries from inbred LCR/Tol and HCR/Tol rats (26–28-weeks-old). In all cases, no more than two males from the same litters were used in the current investigation.

LCR-mt^HCR^/Tol (RGD: 39457682), HCR-mt^LCR^/Tol (RGD: 39457683), LCR/Tol (RGD: 39457699), and HCR/Tol (RGD: 39457701) are new strains that are registered in the RGD. These are named according to the guidelines by the international committee on standardized genetic nomenclature for inbred rats (http://www.informatics.jax.org/mgihome/nomen/strains.shtml#rats). Tol: designates the origin of these rats as Toledo. LCR-mt^HCR^ and LCR-mt^LCR^ are standard nomenclatures for a congenic strain, and in this case, because it is mitochondrial transfer, the name is “conplastic” instead of congenic. Usually, the number of the chromosome follows in the name of the congenic. In this case, it is replaced by the suffix “mt” referring to the transfer of mitochondria.

The full report about conplastic animals is described in our previous study.^15^ Briefly, conplastic animals were developed by taking advantage of the maternal inheritance of mitochondrial genome.^15^ For this, a female HCR progenitor rat was crossed with a male LCR progenitor. The resultant F1 female offspring was backcrossed with male LCR from the contemporaneous inbreeding colony.^15^ This backcross procedure was repeated 17 additional times to generate LCR-mt^HCR^/Tol strain^15^ (inbred). Likewise, a female LCR progenitor was bred with a male HCR progenitor. The F1 female offspring were backcrossed with male HCR from contemporaneous inbreeding colony. This backcross procedure was repeated 17 additional times to generate HCR-mt^LCR^/Tol strain and maintained in subsequent generations by brother–sister mating.^15^ Male LCR-mt^HCR^/Tol and HCR-mt^LCR^/Tol rats were 30–36 weeks old when studied. The conplastic rats were from +30 generations of inbreeding.^15^

We previously^15^ sequenced and compared mtDNA sequence with common inbred strains mtDNA sequences (obtained from GenBank nucleotide database). It was observed that LCR/Tol and HCR-mt^LCR^/Tol mtDNA were identical to the mtDNA reported from Wistar Kyoto inbred strain, while HCR/Tol and LCR-mt^HCR^/Tol mtDNA were identical to the mtDNA reported from Fischer 344 Brown Norway F1-hybrid strain. Sequence differences in mtDNA from conplastic animals are available in our previous study.^15^

All rats were maintained on a 12-h light cycle with normal chow and water ad libitum. All procedures were performed as per NIH guidelines and were in accordance with the guidelines by the Institutional Animal Care and Use Committee of the University of Toledo.

### Echocardiography

Left ventricular (LV) function and geometry were evaluated by echocardiography using a Sequoia C512 System (Siemens Medical) with a 15-MHz linear array transducer as previously described.^18^ Brieﬂy, the rats were anesthetized by mask with 1.5%–2.0% isoﬂurane, had their chests shaved, and were situated in the supine position on a warming pad. After placing electrocardiogram limb electrodes, two-dimensional, two-dimensional guided M-mode and Doppler studies of aortic and transmitral ﬂows were performed from parasternal and foreshortened apical windows. All data were analyzed ofﬂine with the ultrasound system software. Echocardiographic parameters were measured and calculated as previously described.^18^

### Tissue Harvesting

Rats were weighed and euthanized by thoracotomy and exsanguination via cardiac puncture under isoflurane anesthesia (5% in 100% O_2_ administered via nose cone). Mesenteric resistance arteries (MRAs), aortas, mesenteric PVAT (M-PVAT), whole hearts, and tibias were harvested from all rats. The left and right ventricles were dissected and weighed. The following tissues were flash-frozen in liquid nitrogen to be used for molecular biology experiments: MRA, M-PVAT, aortas, and heart.

### Left Ventricle Mass Measurements

It has been shown that body weight correlated nonlinearly (cubically) with tibia length (TL), but linearly with TL^3^ (cubed).^19^ The linear relation with left ventricle (LV) and TL^3^ showed that LV weight crossed the *x*-axis at TL^3^ −26.73 (95% confidence interval (CI) −29.8^3^; −23.4^3^). Therefore, TL and LV weight were indexed by dividing the weights by 26.7^3^ + TL^3^.

### Macroscopic Tissue Imaging

Photographs of M-PVAT were taken prior to vascular function experiments using an iPhone 8 (Apple, Cupertino, CA, USA). The images are presented in Microsoft PowerPoint (Microsoft, Seattle, WA, USA).

### Vascular Function and Mechanics

#### Wire Myograph

Third-order MRAs were mounted on DMT wire myographs (Danish MyoTech, Aarhus, Denmark). The MRAs were normalized to their optimal lumen diameter for active tension development as described previously by our group.^20^ To test vascular smooth muscle cell (VSMC) integrity, the arteries were initially contracted with 120 mmol/L potassium chloride (KCl). To test endothelial integrity, they were contracted with phenylephrine (PE; 3 × 10^−6^ mol/L) and relaxed with acetylcholine (ACh; 3 × 10^−6^ mol/L).

Vasodilation was evaluated by performing cumulative concentration-response curves to ACh and nitric oxide (NO) donor sodium nitroprusside (SNP) after initially contracting them with 3 × 10^−6^ mol/L PE. All curves were from 10^−9^ to 3 × 10^−5^ mol/L. Relaxation responses to ACh and SNP are shown as a percent of the initial PE contraction (3 × 10^−6^ mol/L). Vascular relaxation was also evaluated in the presence of M-PVAT from different strains. For this, 2 cm long strands of M-PVAT were placed inside of each wire myograph chamber prior to the first cumulative concentration-response curve to ACh. Given that we also evaluated the effect of “sandwich” bioassay studies with closely apposed PVAT from LCR with arteries from HCR and vice versa, we deattached the PVAT from all arteries to eliminate divergences in the results between non- and native PVAT. We ensured that the M-PVAT was resting near the mounted artery but was neither compressing the artery nor contacting the force transducers of the wire myograph. Therefore, any changes in the vascular function performed in the presence of PVAT were due to PVAT-derived factors.

#### Pressure Myograph

Fifth to seventh order MRAs were mounted on DMT culture myographs (Danish MyoTech, Aarhus, Denmark) with intraluminal HEPES physiologic buffer and extraluminal Krebs physiologic buffer (in mM: 115 NaCl, 25 NaHCO_3_, 4.7 KCl, 1.2 MgSO_4_.7H_2_O, 2.5 CaCl_2_, 1.2 KH_2_PO_4_, 11.1 glucose, and 0.01 Na_2_EDTA). Intraluminal pressure was raised to 160 mmHg and the artery was adjusted to ensure the walls remained parallel. The MRA was equilibrated at 60 mmHg in normal Krebs for 30 min. After testing VSMC integrity with 120 mmol/L KCl, the extraluminal Krebs was exchanged with high Ca^2+^ Krebs (2.5 mM). Intraluminal pressure was dropped to 3 mmHg and a pressure curve was obtained by increasing the intraluminal pressure in 20 mmHg steps to 160 mmHg. After this curve, the extraluminal buffer was exchanged with zero Ca^2+^ Krebs (plus EGTA) and equilibrated at 60 mmHg for 10 min. After this period, another pressure curve was obtained by increasing the intraluminal pressure in 20 mmHg steps from 3 mmHg to 160 mmHg. Videos and/or images were captured using the DMT data capture software and subsequently analyzed with the VasoTracker Offline Diameter Analyzer for accurate inner and outer diameter measurements.^21^ From the internal and external diameter measurements in the passive conditions, structural parameters were calculated as has been previously described.^22^

### Bioenergetic Assays

VSMCs were harvested from the collected thoracic aortas. Cells were then grown in a humidified chamber at 37°C, with 5% CO_2_, and low glucose Dulbecco’s Modified Eagle’s Medium (GE Healthcare, Logan, UT, USA) containing 10% fetal bovine serum and 1% penicillin/streptomycin solution (Corning, Manassas, VA, USA). In this study, cells of passage four through six were used. We used the Agilent Seahorse XF Cell Mito Stress Test to measure key mitochondrial function parameters by direct measurement of the oxygen consumption rate (OCR) of the VSMCs on the Seahorse XF96 Analyzer. The day prior to the assay, cells were plated at a density of 8000 cells per 80 µL into a 96 well Seahorse XF Cell Culture Microplate and the XF96 sensor cartridge was hydrated in Seahorse XF Calibrant at 37°C in a non-CO_2_ incubator overnight. The plate was mapped to have triplicates per animal. The day of the assay, Seahorse XF DMEM was supplemented with 1 mM pyruvate, 2 mM glutamine, and 10 mM glucose. The final well concentration of oligomycin, an ATP synthase inhibitor, was 1.5 µM, FCCP, an ionophore that shuttles hydrogen ions, was 1 µM, and Rotenone/Antimycin A, an electron transport chain complex I and III inhibitor, respectively, was 0.5 µM. These drugs were added sequentially added by the Seahorse XF96 analyzer to assess mitochondrial respiration. Hoechst 33342 (20 µM) nuclear staining dye was mixed with the Rotenone/Antimycin A mixture to allow for post-assay fluorescent image captures with the BioTek Cytation 5 (BioTek Instruments, Winooski, VT, USA) and subsequent cell counting for data normalization using Fiji,^23^ an image processing focused distribution of ImageJ. We calculated the bioenergetic health index as described previously.^24^

### Statistical Analysis

All statistical analyses were performed using GraphPad Prism 8.2 (La Jolla, CA, USA). Data are presented as mean ± SEM and statistical significance was set at *P* < 0.05. Specific procedures used include Student’s unpaired *t*-test, to compare the means between two samples; one-way or two-way analysis of variance (ANOVA) to compare more than two conditions and concentration-response curves, respectively. Tukey’s post hoc testing and the Bonferroni post hoc testing were used in one-way ANOVA and two-way ANOVAs, respectively. Data are normally distributed. For this, we used normality test (D’Agostino and Person and/or Shapiro–Wilk test). The sample size indicated per experiment is the number of independent rats used.

## Results

### Considerations of the Prior Research Regarding Conplastic Animals and Genetic Divergence Between Strains

To confirm that inbred (Tol) and selectively bred strains preset similar phenotypes, we performed vascular function in MRAs from HCR/Tol and LCR/Tol and compared with selectively bred HCR and LCR. Arteries from inbred (Tol) and selectively bred LCR present with comparable vascular relaxation (Figure S1a). Likewise, arteries from inbred (Tol) and selectively bred HCR present with no differences in acetylcholine-induced relaxation (Figure S1a). However, arteries from LCR, regardless of strain, present with endothelium dysfunction when compared to arteries from inbred and selectively bred HCR (Figure S1a). These data suggest that the genetic divergence between selectively bred and inbred strains did not influence the main phenotype (vascular function) reported in this study (Figure S1a). Supporting these results, body weight was increased in LCR animals regardless the genetic variability (when compared to inbred and selectively bred HCR (Figure S1b and c). Therefore, given that genetic background (inbred vs. selectively bred) is not the major contributing factor of the variation observed in vascular function, specifically in the resistance arteries, we used selectively bred LCR and HCR rats and conplastic Tol animals.

**Figure 1.**
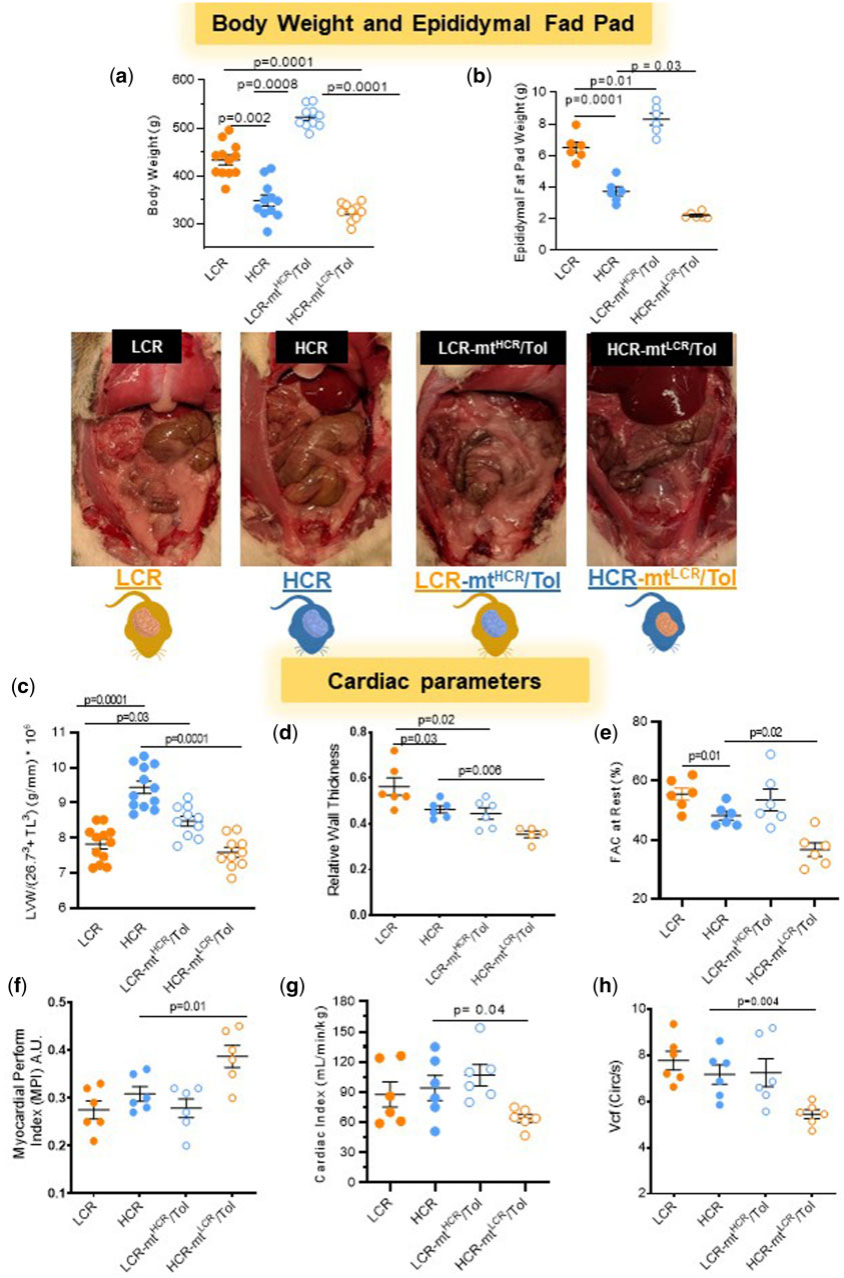
Male LCR and HCR (20–26 Week Old) and Conplastic Animals (Mitochondrial “Swapped” Animals), Male LCR-mt^HCR^/Tol, and HCR-mt^LCR^/Tol (Inbred, *Toledo* Strain; 30–36 Week Old). Variations in intrinsic exercise capacity and interaction between nuclear and mtDNA affect adiposity and cardiac structure and function. (**A**) Body weight (g) (*n* = 10–12, one-way ANOVA). (**B**) Epididymal fat pad weight (*n* = 6 per group, one-way ANOVA and Tukey’s post hoc tests) and representative images of the exposed abdomen of each cohort. (**C**) Left ventricular weight (g) normalized by 26.7^3^ + TL^3^ (mm) (LCR and HCR [*n* = 12]; HCR-mt^LCR^/Tol and LCR-mt^HCR^/Tol [*n* = 10]; one-way ANOVA). Echocardiography measurements at rest: (**D**) RWT; (**E**) FCA; (**F**) MPI; (**G**) CI; and (**H**) Vcf (*n* = 5–6; one-way ANOVA, Tukey’s post hoc tests and unpaired *t*-test).

### Metabolic and Cardiac Parameters

LCR rats presented with higher body weight compared to HCR rats (Figure 1A). Mitochondrial swap increased body weight in LCR-mt^HCR^/Tol rats when compared to LCR (Figure 1A). There were no significant differences in body weight between HCR and HCR-mt^LCR^/Tol *Tol* rats (Figure 1A). Interaction between nuclear and mtDNA increased abdominal and epididymal fat pad weight in LCR-mt^HCR^/Tol (Figure 1B). On the contrary, interaction between nuclear and mtDNA decreased abdominal and epididymal fat pad weight in HCR-mt^LCR^/Tol (Figure 1B).

Regarding cardiac structure and function, HCR presents higher LV mass when compared to LCR (Figure 1C). Interestingly, mitochondrial swap increased LV mass in LCR-mt^HCR^/Tol compared to LCR (Figure 1C). On the contrary, HCR-mt^LCR^/Tol lowered this parameter (Figure 1C). Echocardiography showed that relative wall thickness (RWT) and fractional area change (FAC), which estimates LV systolic function are lower in HCR compared to LCR (Figure 1D and E). No differences were observed in the myocardial performance index (MPI), cardiac index (CI), a measure of cardiac output controlled for body weight differences, or velocity of circumferential fiber shortening (Vcf), an index for myocardial contractility, between LCR and HCR (Figure F–H). However, HCR-mt^LCR^/Tol presents with a significant decrease in RWT, FAC, CI, and Vcf (Figure 1D, E, G, and H) and an increase in MPI (Figure 1F). No significant changes were observed in FAC, CI, MPI, and Vcf (Figure 1E–H) in LCR-mt^HCR^/Tol, but decreased RWT (Figure 1D).

### Vascular Function, Mechanics, and Structure

**Figure 2.**
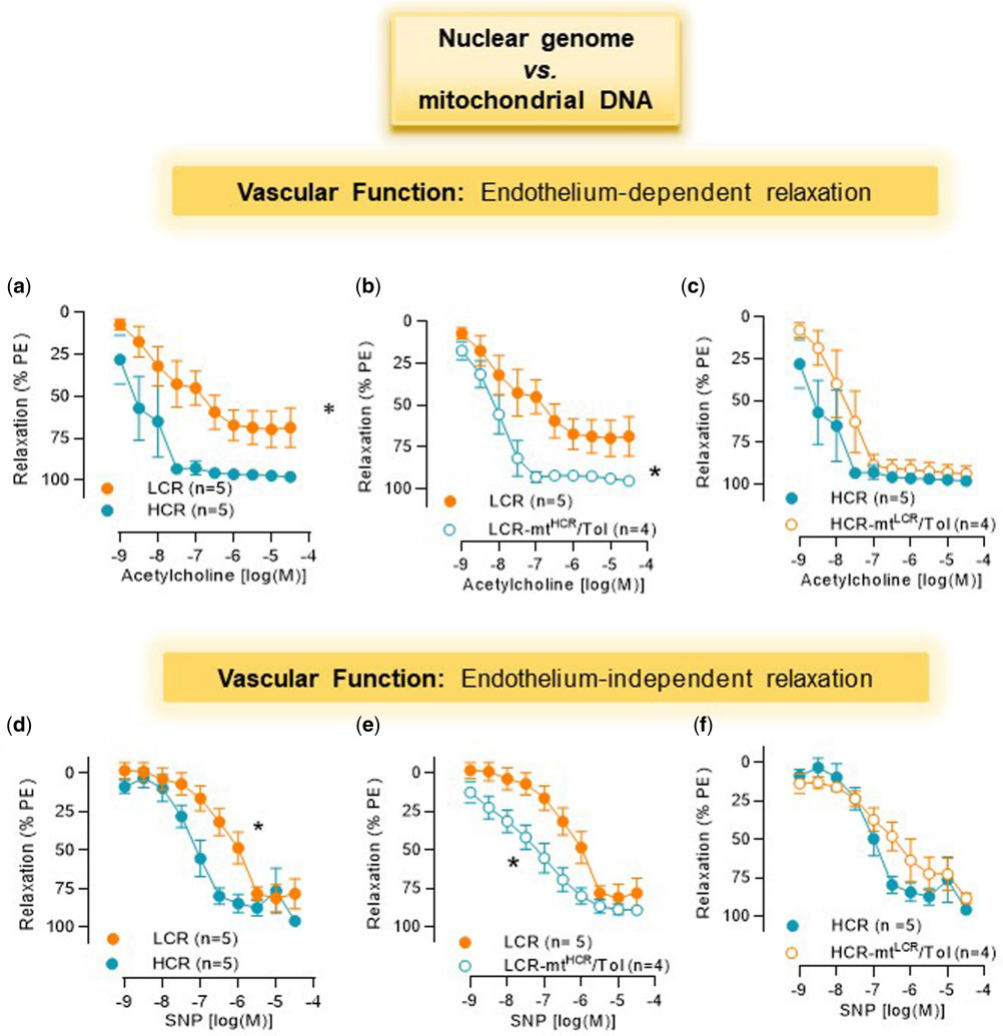
Concentration-Response Curves to Acetylcholine (**A–C**) and SNP (**D–F**) in MRAs from Male LCR and HCR (20–26 Week Old) and Conplastic Animals (Mitochondrial “Swapped” Animals), Male LCR-mt^HCR^/Tol and HCR-mt^LCR^/Tol (Inbred, *Toledo* Strain; 30–36 Week Old). Arteries were contracted with PE 3 × 10^−6^ M. Number of animals are indicated in the graphs. Data are presented as mean ± SEM. Two-way ANOVA and Bonferroni post hoc testing were used. *P < 0.05.

LCR presents with decreased endothelium-dependent and independent relaxation in resistance arteries when compared to HCR (Figure 2A and D). On the contrary, LCR-mt^HCR^/Tol led to an improvement in these responses (Figure 2B and E). There were, however, no significant differences in acetylcholine and SNP-induced relaxation in arteries from HCR and HCR-mt^LCR^/Tol rats (Figure 2C and F).

**Figure 3.**
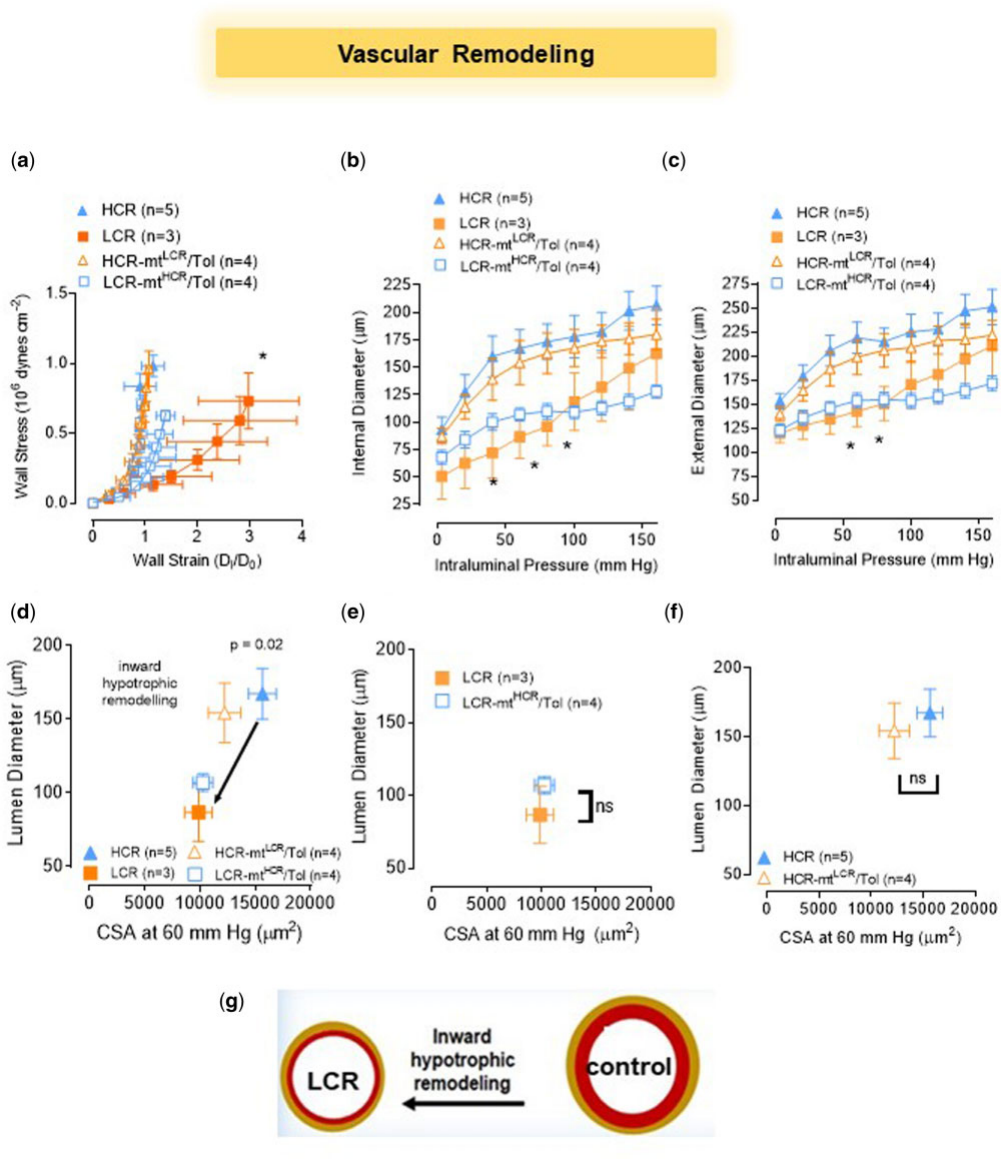
Male LCR and HCR (20–26 Week Old) and Conplastic Animals (Mitochondrial “Swapped” Animals), Male LCR-mt^HCR^/Tol, and HCR-mt^LCR^/Tol (Inbred, *Toledo* Strain; 30–36 Week Old). Structural characteristics of fifth to seventh order MRAs are dependent upon variations in intrinsic exercise capacity but independent of mtDNA. (**A**) MRA wall stress–strain plot, *P < 0.0001 (nonlinear regression). Intraluminal pressure curves for (**B**) internal diameter and (**C**) external diameter (two-way ANOVA and Bonferroni post hoc tests were used. *P < 0.05). Vascular remodeling plots of the lumen diameter vs. cross*-*sectional area at 60 mm Hg where (**D**) LCR vs. HCR, *P* = 0.02, (**e**) LCR-mt^HCR^/Tol vs. LCR, and (**F**) HCR-mt^LCR^/Tol vs. HCR (unpaired *t*-test). (**G**) Scheme shows that LCR presents inward hypotrophic remodeling.

Pressure myography enables us to examine the passive structural characteristics of fifth to seventh order MRAs. Figure 3A plots the calculated wall stress and wall strain against each other showing a difference in elasticity of LCR compared to HCR. However, plotting the internal (Figure 3B) and external (Figure 3C) diameter in an intraluminal pressure curve reveals LCR MRA to be consistently smaller than that of HCR. Therefore, we classified LCR MRA vascular remodeling as inward hypotrophic compared to HCR (Figure 3D and G). Interaction between nuclear and mtDNA did not change vascular remodeling of LCR-mt^HCR^/Tol (Figure 3A–E) or HCR-mt^LCR^/Tol (Figure 3A–D and F). Wall thickness (WT) was also consistent between groups (WT [µm] at 60 mmHg: LCR: 28.0 ± 3.0; HCR: 26.0 ± 1.3; LCR-mt^HCR^/Tol: 23.2 ± 0.8; HCR-mt^LCR^/Tol: 22.4 ± 2.2).

**Figure 4.**
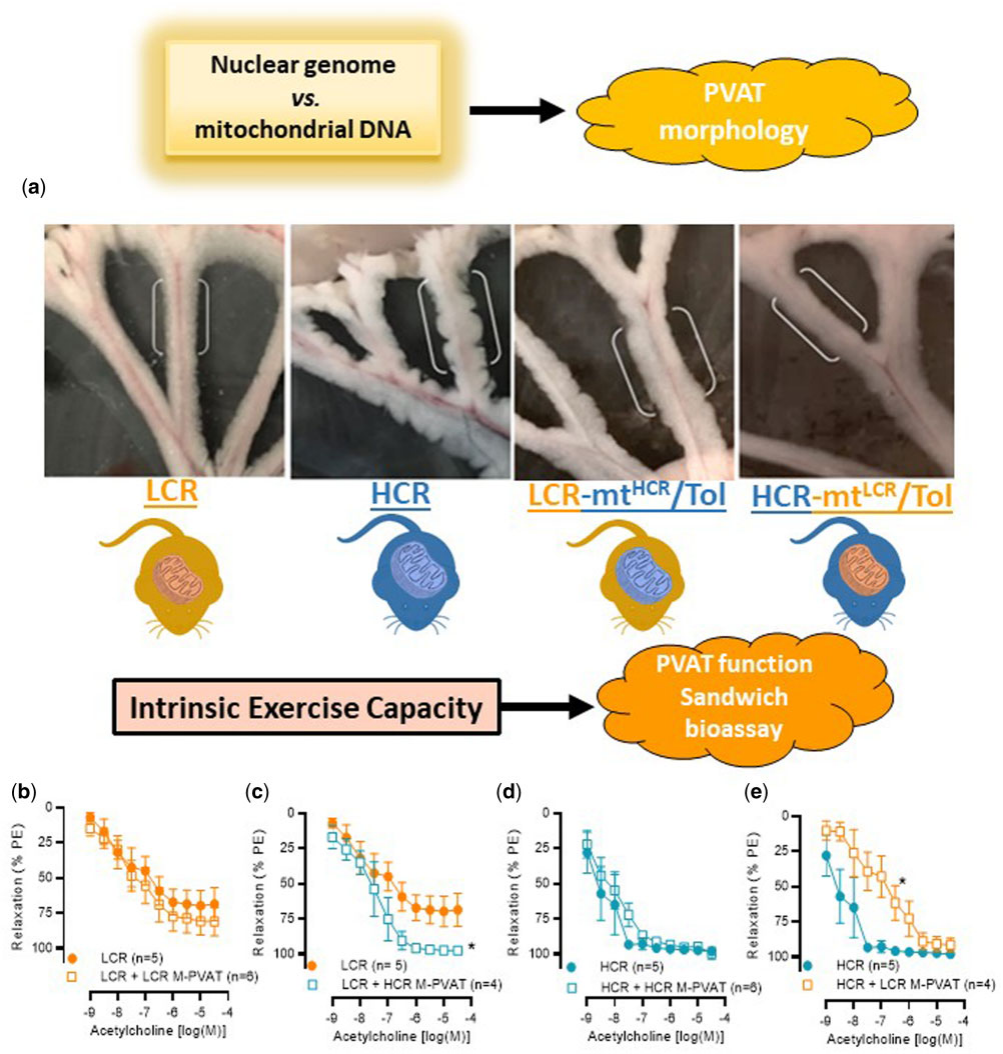
PVAT Morphology and Function. (**A**) Representative images of perivascular adipose tissue from MRAs (M-PVAT) of each cohort: male LCR and HCR (20–26 week old) and male LCR-mt^HCR^/Tol and HCR-mt^LCR^/Tol (inbred, Toledo strain; 30–36 week old). Concentration response curves to acetylcholine in arteries contracted with PE 3 × 10^−6^ M in the presence or absence of native or exchanged (sandwich bioassay) M-PVAT. (**B**) Dysfunctional LCR M-PVAT did not improve vasodilation in arteries from LCR. (**C**) “Sandwich bioassay” shows that HCR M-PVAT improved vasodilation in arteries from LCR. (**D**) Arteries from HCR in the presence of native M-PVAT. (**E**) LCR M-PVAT decreased acetylcholine-induced vasodilation in arteries from HCR. A number of animals are indicated in the graphs. Data are presented as mean ± SEM. Two-way ANOVA and Bonferroni post hoc tests were used. *P < 0.05.

During the isolation of MRA from these animals, we observed a ridged, bulbous appearance of HCR M-PVAT when compared to any other group (Figure 4A). However, this phenomenon was observed in a subset of animals (6 out 11 [55%]). Interestingly, this change in morphology was not present in HCR-mt^LCR^/Tol and LCR-mt^HCR^/Tol (Figure 4A). As M-PVAT is a well-established modulator of vascular function and, in physiological conditions, has a vasodilatory and/or anticontractile effect, we questioned if function would follow form. We observed that native LCR M-PVAT did not improve relaxation (Figure 4B). Interestingly, LCR arteries that received an HCR M-PVAT via “sandwich” bioassay presented a significantly improved in acetylcholine-induced relaxation (Figure 4C). Native HCR M-PVAT did not present change in acetylcholine-mediated relaxation response (Figure 4D). On the contrary, HCR arteries that received an LCR M-PVAT (sandwich bioassay studies) presented with a significant reduction in vasodilation (Figure 4E).

### Bioenergetic Profiling

**Figure 5.**
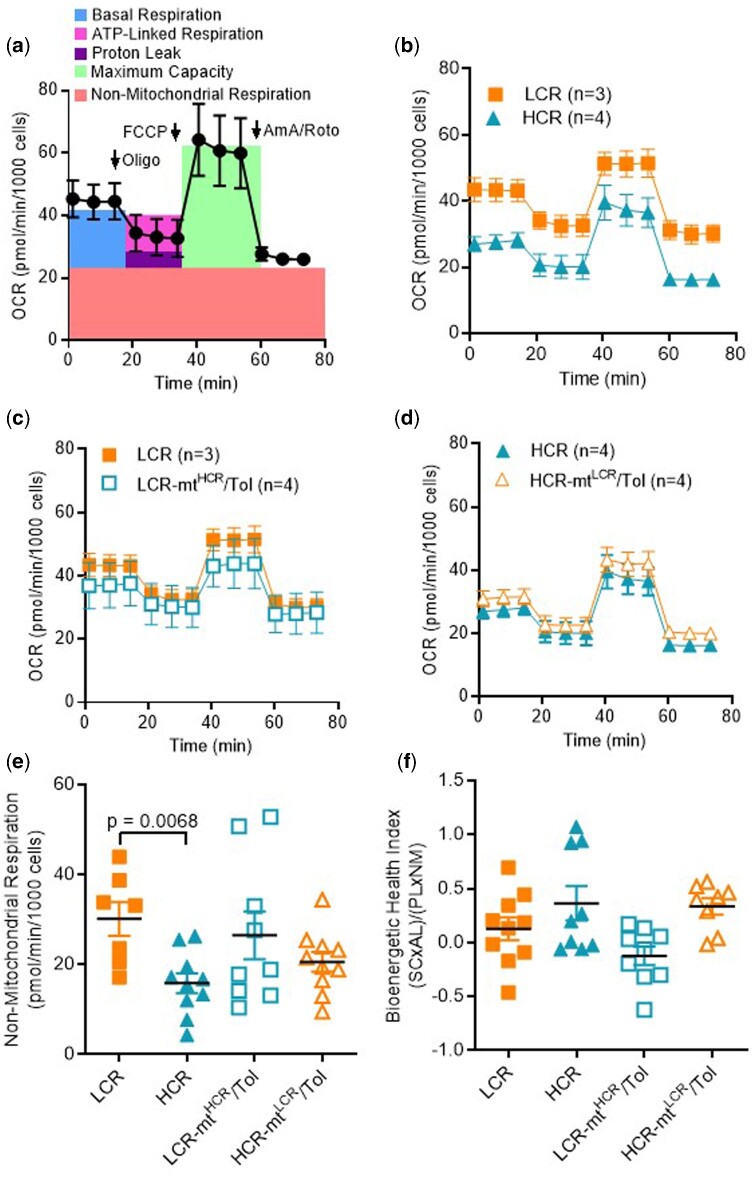
Male LCR and HCR (20–26 Week Old) and Conplastic Animals (Mitochondrial “Swapped” Animals), Male LCR-mt^HCR^/Tol, and HCR-mt^LCR^/Tol (Inbred, *Toledo* Strain; 30–36 Week Old). (**A**) Standard scheme for the mitochondrial stress test using control VSMCs where OCR is normalized by cell count and evaluated over time. Oligomycin (Oligo), mitochondrial phosphorylation uncoupler (FCCP), and Antimycin A/Rotenone (AmA/Roto) are administered to evaluate key parameters. OCR plots for (**B**) LCR vs. HCR (**C**) LCR vs. LCR-mt^HCR^/Tol and (**D**) HCR vs. HCR-mt^LCR^/Tol. A number of animals are indicated in the graphs. (**E**) The calculated parameter nonmitochondrial respiration and (**F**) the calculated bioenergetic health index. In the Figures (**B–D**), the data are presented as mean ± SEM HCR (*n* = 4), LCR (*n* = 3), LCR-mt^HCR^/Tol (*n* = 4), and HCR-mt^LCR^/Tol (*n* = 4). In Figures (**E** and **F**), the data presented are the individual results HCR (*n* = 10), LCR (*n* = 7), LCR-mt^HCR^/Tol (*n* = 9), and HCR-mt^LCR^/Tol (*n* = 10). (One-way ANOVA and Tukey’s post hoc tests were used.)

Given that interaction between nuclear and mtDNA plays a role in vascular function, we aimed to further understand the mitochondrial function of VSMCs. Figure 5A represents a scheme showing the overall bioenergetics assay using VSMCs from rats not selectively bred for low and high maximal treadmill-running distance (control). No differences were observed in proton leak, maximum capacity, and basal and ATP-linked respiration between LCR and HCR (Figure 5B). However, we observed that VSMCs from LCR presented with a significant increase in nonmitochondrial respiration as evident by a curve that was upwards compared to that of HCR (Figure 5B and E). Interaction between nuclear and mtDNA did not change these parameters in LCR-mt^HCR^/Tol or HCR-mt^LCR^/Tol (Figure 5C and D). No substantial difference was observed in bioenergetic health index between groups (Figure 5F).

## Discussion

In animals, inherited and somatic genetic variation in the mitochondrial genome play a fundamental role in the phenotypic variation.^25^ It has been shown that the variation in mitochondrial membrane potential is an important determinant of phenotypic heterogeneity, gene expression, stress tolerance, and drug resistance in individual cells.^25^ The maternal inheritance of animal mtDNAs is highly concerted with specific systems that actively exclude the paternal mitochondria and mtDNAs during fertilization.^26^ The paternal mtDNA is excluded because incompatibility could occur if two normal but different mtDNAs are mixed within the same animal. Accordingly, it has been shown that the heteroplasmy of mtDNA is genetically unstable and results in altered behavior and cognition.^27^ These observations indicate that reducing heteroplasmy in patients with mtDNA mutations could be a therapeutic objective.^28^ Previously, we sequenced and compared mtDNA sequence with common inbred strains mtDNA sequences.^15^ LCR and HCR-mt^LCR^/Tol mtDNA were identical to the mtDNA reported from Wistar Kyoto inbred strain, while HCR and LCR-mt^HCR^/Tol mtDNA were identical to the mtDNA reported from Fischer 344 Brown Norway F1-hybrid strain. In this work, we sought to understand the influence and mechanisms of inherited exercise capacity and normal, but swapped, mitochondria on vascular physiology in untrained animals.

The major findings of this study are that deleterious vascular plasticity in resistance arteries is present in LCR, while HCR has beneficial vascular plasticity. Inheritance of mtDNA from a mother with high intrinsic exercise capacity combined with the composite nuclear DNA of that mother and a father with low intrinsic exercise capacity results in offspring with improved vascular function. On the contrary, mtDNA of mothers with low intrinsic exercise capacity did not change this parameter, but lead to a dilated cardiomyopathy phenotype in HCR animals.

An “athlete’s heart” is a concept that has been extensively used to characterize the changes that occur to cardiac function and structure due to long-term physical exercise.^29^ Specifically, chronic physical exercise may lead to physiological cardiac remodeling and hypertrophy that occurs as an adaptive response.^29^ Aerobic exercise training, such as long-distance running or swimming, may lead to increased cardiac mass with LV chamber dilation, known as eccentric hypertrophy.^29^ Interesting, it has been shown that right ventricle (RV) systolic function in athletes does not usually differ from nonathletes. However, in highly trained endurance athletes where the RV dilation is prominent, a slight reduction in RV global systolic function assessed by RV FAC may be shown.^30^^,^^31^ On the contrary, resistance training, such as weight lifting and wrestling, may lead to increased cardiac mass without LV chamber dilation, referred to as concentric hypertrophy.^29^ In this study, we used untrained animals to understand the influence of innate aerobic capacity on the cardiovascular system. However, similar to eccentric hypertrophy induced by regular aerobic exercise, untrained HCR hearts demonstrate increased LV mass and reduced RWT when compared to LCR counterparts. In addition, interaction between nuclear and mtDNA significantly increased cardiac mass and decreased RWT in LCR-mt^HCR^/Tol, mimicking eccentric hypertrophic changes. These data suggest that at least some of the physiological adaptations in the heart due to exercise are also heritable traits. Research by Hoppel’s group has established that mitochondrial dysfunction is implicated in the development of pathologic cardiac hypertrophy and heart failure.^32^ Although a reduction in LV mass was observed in the HCR-mt^LCR^/Tol group compared to the HCR, this change was accompanied by reduced LV function (increased MPI and reduced Vcf, FAC, and CI) and decreased RWT, reflecting changes similar to those seen in the development of dilated cardiomyopathy. These results suggest that mtDNA is indeed a heritable and important factor in determining cardiac phenotype and function.

Changes in the body weight from these animals were directly correlated with changes in epididymal fat pad. Epididymal fat is a visceral fat and excess of this fat brings on a host of consequences including increases in cardiovascular and metabolic disease risk.^33^ What is peculiar is that LCR mtDNA in HCR further reduced the amount of visceral fat while HCR mtDNA in LCR further increased the amount of visceral fat. Although, we do not know why this phenotype was observed in these animals, the increase and decrease in body weight of LCR and HCR and LCR-mt^HCR^/Tol and HCR-mt^LCR^/Tol may not be associated with food intake. Accordingly, it has been shown that lean, HCR rats consumed more calories after mass correction compared to overweight, LCR rats.^34^ Thus, the lean phenotype is not characterized by low caloric intake in rats. These authors suggested that leanness and high physical activity levels may have resulted as a byproduct during natural selection of high capacity for running endurance.^34^

As described in our previous study,^15^ LCR animals presented with a slight elevation in blood pressure when compared to HCR and that mismatched mitochondria decreased blood pressure in LCR-mt^HCR^/Tol. Given that resistance arteries are important to control blood pressure, here we investigated if deleterious functional and structural changes would be present in arteries from LCR and if LCR-mt^HCR^/Tol would improve these parameters. We observed that LCR MRA presented a significant dysfunction in the endothelium-induced relaxation when compared to HCR. Endothelial dysfunction was also correlated with mtDNA, since LCR-mt^HCR^/Tol restored relaxation response of LCR MRA. These findings suggest that mtDNA is important to maintain vascular function and subsequently, blood pressure homeostasis. Mitochondria within VSMC are implicated in maintaining vascular tone and energy production for vascular cell secretion.^35–37^ Given that LCR-mt^HCR^/Tol also had improved endothelium-independent relaxation, it is possible to suggest that the restoration in vascular relaxation is, at least in part, mediated by intrinsic changes in VSMCs. In this study, mitochondrial function, measured by OCR of the VSMCs, was not different between LCR and HCR. However, we observed that nonmitochondrial respiration was elevated in LCR. In leucocytes, nonmitochondrial OCR is typically attributed to enzymes associated with inflammation, including cyclooxygenases, lipoxygenases, and NADPH oxidases, and are regarded as negative indicators of bioenergetic health. Therefore, an increase in nonmitochondrial respiration is due to an increase in reactive oxygen species generation. In the last decade, the importance of bioenergetic health has increased, suggesting that the measurement could become the next major predictor of mortality and disease, like BMI.^24^ Therefore, it is surprising that we did not observe changes in this parameter between LCR and HCR. The lack of that change in the index and in the overall bioenergetic profile collected may be because of contributions from elsewhere other than the mitochondria.

Examining passive, mechanical properties of MRA led us to apply the label of “inward hypotrophic remodeling” (vascular atrophy) to both the LCR and LCR-mt^HCR^/Tol rats.^8^ In this form of vascular remodeling, the MRA is associated with a reduction in flow.^38^ This type of remodeling has been previously observed in vessels such as the renal afferent arterioles of spontaneously hypertensive rats.^39^ In arteries with remodeling or changes in myogenic tone, the subsequent turbulent flow can cause endothelial dysfunction.^40^ This correlates with the endothelial dysfunction that is present in LCR MRA. However, a causative relationship between vascular remodeling and endothelial dysfunction was not assessed in this study and will be the subject of future studies. Chronic changes in hemodynamic forces structurally alter the vascular wall and vice versa. One important concept of vascular mechanics is that proportional composition of blood vessels influences passive distensibility of the vessel wall. The concept considers the relation between structure and mechanics of the vessel wall in terms of the elastic moduli of individual wall components. In an interesting study, Baumbach et al.^41^ showed that in hypertension, one mechanism that may protect cerebral vessels is increasing in passive distensibility.^41^ It was suggested that increases in passive distensibility may increase the effectiveness of autoregulation of blood flow. Likewise, in this study, we found that arteries from LCR rats presented with a rightward shift in the stress–strain curve, suggesting that these arteries are more compliant. It is possible that reduction in the stiffness is due to a lower wall tension and flow seen in arteries with hypotrophic remodeling. Additional studies into the mechanisms of this vascular remodeling and its significance needs to be explored.

In this study, we observed macrostructural change of M-PVAT in HCR animals. Specifically, HCR M-PVAT presented a ridged and bulbous appearance when compared to LCR. These changes could be due to mtDNA, since LCR-mt^HCR^/Tol presented a PVAT with ridged appearance while HCR-mt^LCR^/Tol abolished the ridged appearance of HCR PVAT. The macrostructure of M-PVAT is rarely mentioned in the current literature because typically, small sections of PVAT or PVAT supernatant are used to study the biochemical characteristics of the tissue. Presently, there is no standard method for quantifying visible differences in PVAT macrostructure. We still do not know why these morphology changes occurred in HCR M-PVAT. However, it is possible to infer that with a greater surface area, the HCR M-PVAT can exert a greater net vasodilatory influence via increased paracrine communication between the M-PVAT, MRA, and the surrounding environment.

In health, PVAT secretes anticontractile factors that relax the underlying artery.^42^ However, PVAT dysfunction paired with endothelial dysfunction contributes to cardiovascular disease.^43^ Therefore, our finding that LCR M-PVAT is dysfunctional lends further support to prior research that low intrinsic exercise capacity correlates with a predisposition to vascular disease risk.^6^^,^^7^^,^^10^ The respective balance of contribution between the dysfunctional MRA and the dysfunctional M-PVAT to these disease factors is unclear. However, it has been shown that PVAT dysfunction intensified adventitial remodeling in obese/metabolic syndrome induced mini pigs.^44^ Interestingly, we observed that the presence of HCR M-PVAT had a restorative impact on LCR MRA relaxation to acetylcholine. Adipose tissue transplantation as a therapeutic is a developing field of study. Recently, the transplantation of normal adipose tissue was demonstrated to improve blood flow and reduced inflammation in high-fat diet-fed mice with hind limb ischemia.^45^ Further, abdominal aortic perivascular adipose tissue transplantation found that dysfunctional PVAT could have remote effects such as endothelial dysfunction and the augmentation of atherosclerosis.^46^ Since we observed that M-PVAT from LCR-mt^HCR^/Tol restored endothelial cell-mediated impaired relaxation in LCR MRA, there is promise in further examining the characteristics of HCR M-PVAT and its potential therapeutic capabilities in vascular diseases. A limitation of this study was that the specific adipose-derived contracting or relaxation factors were not investigated. The identification of these factors is fundamental for understanding mechanisms of vascular plasticity in divergent models of untrained intrinsic exercise capacity.

In conclusion, our results are the first to demonstrate that the interplay between the nuclear genome and the maternally inherited mitochondrial genome with high intrinsic exercise capacity decreases vascular dysfunction in the offspring, while mitochondria DNA of mothers with low intrinsic exercise capacity increases vascular risk.

## Limitations of the Study

By comparing inbred LCR-mt^HCR^/Tol and HCR-mt^LCR^/Tol to their outbred progenitors, there is a chance to reduce power and increase variation in the experimental models due to high levels of genetic variation. Such comparisons are not equal to comparisons of conplastic strains because these outbred parental strains and conplastic strains differ in many genes in nuclear DNA in addition to mtDNA, especially when outbred LCR and HCR strains were bred by a system that minimized inbreeding.^4^ On the contrary, these experimental results may be more applicable to natural or human populations. Further, Tuttle et al.^47^ published an elegant study in *Nature Methods* comparing phenotypic variation between inbred and outbred animals. Traditionally, inbred mice are preferred over outbred mice because it is assumed that they display less trait variability. However, Tuttle et al.^47^ compared coefficients of variation and they did not find evidence of greater trait stability in inbred mice. They concluded that contrary to conventional wisdom, outbred mice might be better subjects for most biomedical research.

In a previous study, Houštěk et al.^48^ observed that conplastic strains that were genetically identical except for their mtDNA, SHR-mt^F344^ vs. SHR, presented multiple differences in the mitochondria genome.^48^ Accordingly, they observed that F344 vs. SHR mtDNA presented nonsynonymous substitutions in protein-coding genes, as well as mutations in tRNA and rRNA genes that were associated with reduced cardiac oxidative phosphorylation system enzyme activity and cardiac hypertrophy with systolic dysfunction that was independent of blood pressure.^48^ This study provided evidence that inherited alterations in mitochondrial genome, in the absence of variation in the nuclear genome and other confounding factors, predispose to cardiac hypertrophy and systolic dysfunction.^48^ Previously, we observed that LCR/Tol and HCR-mt^LCR^/Tol mtDNA were identical to the mtDNA reported from Wistar Kyoto inbred strain, while HCR/Tol and LCR-mt^HCR^/Tol mtDNA were identical to the mtDNA reported from Fischer 344 Brown Norway F1-hybrid strain.^15^ There is a possibility that mtDNA differs in multiple nonsynonymous mutations in protein-coding genes as well as in rRNA and tRNA genes as suggested by Houštěk et al.^48^

We also would like to highlight that we used a small group size (*n* = 3) for the LCR arteries to evaluate vascular mechanics (Figure 3). The reason for this issue was because we lost two arteries (lumen diameter <50 μm) due to technical difficulties in the experimental preparation. Despite this low sample number, there was a significant difference in the strain and stress measurements between LCR vs. HCR. There were no differences between the swapped strains (LCR vs. LCR-mt^HCR^/Tol and HCR vs. HCR-mt^LCR^/Tol) (Figure 3E and F).

## Supplementary Material

zqaa029_Supplementary_Data
