# Supplementary material for: Intrinsic Exercise Capacity and Mitochondrial DNA Lead to Opposing Vascular-Associated Risks
Source: Function (Oxf). 2020 Nov 3;2(1):zqaa029. doi: 10.1093/function/zqaa029 (PMC7749784; doi:10.1093/function/zqaa029)
Supplement: zqaa029_Supplementary_Data [file zqaa029_supplementary_data.docx]

**Supplementary Material**

Intrinsic exercise capacity and mitochondrial DNA lead to opposing vascular-associated risks

Shaunak Roy1, Jonnelle M Edwards1, Jeremy C Tomcho1, Zachary Schreckenberger1, Nicole R Bearss1, Youjie Zhang1, Eric E Morgan1,2, Xi Cheng1, Adam C Spegele1, Matam Vijay-Kumar1, Cameron G McCarthy1, Lauren G Koch1, Bina Joe1 and Camilla Ferreira Wenceslau1*

Department of Pharmacology and Physiology, University of Toledo College of Medicine and Life Science^s1^; Department of Radiology Nationwide Children's Hospital^2^, OH, USA

*Corresponding Author:

Camilla Ferreira Wenceslau, Ph.D.

Assistant Professor

Laboratory of Vascular Biology (LVB)

Department of Physiology and Pharmacology

University of Toledo College of Medicine & Life Sciences

http://www.utoledo.edu/med/depts/physpharm/faculty/camillawenceslau.html

3000 Transverse Drive

Toledo, Ohio 43614-2598

Phone#: 419.383.5307

Camilla.Wenceslau@utoledo.edu
